# α-glucosidase and glycation inhibitory effects of *costus speciosus* leaves

**DOI:** 10.1186/s12906-015-0982-z

**Published:** 2016-01-05

**Authors:** Handunge Kumudu Irani Perera, Walgama Kankanamlage Vindhya Kalpani Premadasa, Jeyakumaran Poongunran

**Affiliations:** 1 Department of Biochemistry, Faculty of Medicine, University of Peradeniya, Peradeniya, Sri Lanka; 2 Postgraduate Institute of Science, University of Peradeniya, Peradeniya, Sri Lanka

**Keywords:** *Costus speciosus* leaf, α-amylase, α-glucosidase, Glycation, Inhibitors

## Abstract

**Background:**

Hyperglycaemia is a salient feature of poorly controlled diabetes mellitus. Rate of protein glycation is increased with hyperglycaemia leading to long term complications of diabetes. One approach of controlling blood glucose in diabetes targets at reducing the postprandial spikes of blood glucose. The objectives of this study were to assess the in vitro inhibitory effects of *Costus speciosus* (COS) leaves on α-amylase and α-glucosidase activities, fructosamine formation, protein glycation and glycation-induced protein cross-linking.

**Methods:**

Methanol extracts of COS leaves were used. Inhibitory effects on enzyme activities were measured using porcine pancreatic α-amylase and α-glucosidase from *Saccharomyces cerevisiae* in the presence of COS extract. Percentage inhibition of the enzymes and the IC_50_ values were determined. In vitro protein glycation inhibitory effect of COS leaves on early and late glycation products were measured using bovine serum albumin or chicken egg lysozyme with fructose. Nitroblue tetrazolium was used to assess the relative concentration of fructosamine and polyacrylamide gel electrophoresis was used to assess the degree of glycation and protein cross-linking in the reaction mixtures.

**Results:**

α-Glucosidase inhibitory activity was detected in COS leaves with a IC_50_ of 67.5 μg/ml which was significantly lower than the IC_50_ value of Acarbose (*p* < 0.01). Amylase inhibitory effects occurred at a comparatively higher concentration of extract with a IC_50_ of 5.88 mg/ml which was significantly higher than the IC_50_ value of Acarbose (*p* < 0.01). COS (250 μg/ml) demonstrated inhibitory effects on fructosamine formation and glycation induced protein cross-linking which were in par with 1 mg/ml aminoguanidine were detected.

**Conclusion:**

Methanol extracts of COS leaves demonstrated in vitro inhibitory activities on α-glucosidase, fructosamine formation, glycation and glycation induced protein cross-linking.

These findings provide scientific evidence to support the use of COS leaves for hypoglycemic effects with an added advantage in slowing down protein glycation.

## Background

Diabetes mellitus is a chronic disease which causes millions of deaths worldwide each year as a result of the associated complications [1]. Persistently elevated blood glucose concentration is a salient feature of poorly controlled diabetes. As a result, protein glycation is commenced with the non-enzymatic addition of sugar molecules into proteins at an accelerated speed, as the rate of this process depends on the concentration of sugar. In the early stages of glycation, the sugar reacts with free amino groups of proteins, to form stable Amadori products such as fructosamine [2]. Glycation proceeds over a period of time which leads to the production of advance glycation end products (AGEs). AGEs cause irreversible structural and functional damage to the affected molecules [3]. Protein cross-linking occurs at the later part of glycation, further aggravating the tissue damage especially when the cross-links are formed in long-lived proteins, such as collagen [4]. Protein glycation is identified as a primary cause for the development of chronic diabetic complications such as retinopathy, nephropathy and cardio vascular diseases [5]. Glycation induced cross-linking cause extra cellular matrix proteins rigid and less susceptible to enzymatic digestion. This leads to thickening of basement membranes affecting organ functions as observed in diabetic nephropathy [6]. Furthermore the role of AGEs has been discussed on aging with a particular emphasis on skin aging [7] and age related neurodegenerative diseases [8].

Therapeutic agents used for diabetes, target to bring down the blood glucose concentrations as close as to normal physiological levels [9]. Some antidiabetic drugs target key enzymes hydrolyzing the carbohydrates such as α-amylase and α-glucosidase in order to decrease the post-prandial elevation of blood glucose [10, 11]. α-Amylase hydrolyses the initial hydrolysis of starch into α-limit dextrins, maltose and maltotriose [12]. α-Glucosidase catalyzes the release of absorbable monosaccharides from the substrate [13]. As a result, postprandial spikes of blood glucose appear during the digestion of dietary starch. Inhibition of α-amylase and α-glucosidase delays carbohydrate digestion and decrease glucose absorption bringing down the post-prandial elevation of blood glucose. Inhibition of protein glycation is another therapeutic approach which can delay the progression of diabetic complications. However, the synthetic drugs which act as inhibitors of amylase, glucosidase and glycation show side effects in addition to the desirable effects [3, 11].

Natural remedies used since ancient times became popular as effective, inexpensive and safe mode of treating diabetes [14]. It is recognized that there are more than 1,200 species of plants with hypoglycemic activity [15]. A review on the medicinal plants used to treat diabetes by ayurvedic and traditional physicians in Sri Lanka has reported the use of approximately 126 antidiabetic plants including *Costus speciosus* leaves [16]. However, most of these are used in traditional practice without a proper scientific scrutiny [17].

*Costus speciosus* (COS) or *Cheilocostus speciosus* is used to treat various diseases and are used as an ornamental plant too [18]. It belongs to the family Costaceae (Zingiberaceae). The genus Costus consists of approximately 175 species [19]. COS is a plant that is known as *Thebu* in Sinhala and crepe ginger or spiral ginger in English. Leaves of COS are arranged spirally around the trunk. Rhizome of COS is reported to possess hypoglycemic properties. Leaves of COS are popular among Sri Lankans which are included in the main meals as a salad [20–22]. Consumption of COS leaves are believed to be effective in controlling the blood glucose and lipid levels [21, 23]. A recent study conducted in Sri Lanka has shown that the usage of herbal medicines is 76 % among a group of 252 type 2 diabetic patients investigated who were on one or more oral hypoglycaemic agents [24]. Among them 47 % have consumed COS leaf as a salad in their main meals [24]. It is known that diabetic patients eat one leaf daily in India to keep the blood glucose concentration low [25]. COS was among three commonly used (>20 % usage) plants to lower blood glucose concentration by the Puerto Rican population [26]. Remedies prepared from two plants including COS are commonly known as “insulin” by the studied population in Puerto Rican [26]. When investigated, it was recognized that a daily dosage of approximately 0.8 of a COS leaf (~2.5 g fresh leaf) is consumed [26].

Several investigations have proven the hypoglycaemic effects of COS rhizome in alloxan or streptozotocin induced diabetic rats [27–30]. However, evidence to prove the effectiveness of COS leaf are lacking. Furthermore there are no reports on the antiglycation potential of COS leaves as per up to date literature. The objective of this study was to assess the in vitro inhibitory effects of *Costus speciosus* leaves on α-amylase and α-glucosidase activities, fructosamine formation, protein glycation and glycation-induced protein cross-linking.

## Methods

### Plant parts

Leaves of *Costus speciosus* (Koenig) Smith (Family Costaceae) were collected in March 2013 from Moratuwa, Sri Lanka, authenticated by the Deputy Director/National Herbarium and the voucher samples (Voucher No. HKIP-SLS-BIO-2013-02) were deposited at the National Herbarium, Royal Botanical Gardens, Peradeniya, Sri Lanka.

### Preparation of methanol extracts

COS leaves were collected, cleaned and dried under shade for approximately 10 days. Dry leaves were ground using an electric grinder. Dry powder (10 g) of COS leaves was extracted three times with methanol (100 ml) using the sonicator. Filtered methanol was evaporated using the rotary evaporator (Buchi RII) at a temperature below 50 °C [31]. Dry form of the crude methanol was resuspended in phosphate buffer (pH 7.4) to the required working concentrations prior to the experiments.

### Measurement of α-Amylase inhibitory effect of COS leaves

α-Amylase inhibitory effect of COS extract was assessed using the pre-incubation method as described by Geethalakshmi et al. [32] from the method adapted from Bernfeld [33]. Porcine pancreatic α-amylase (Sigma) in ice-cold distilled water (5 unit/ml solution) and potato starch (1 % w/v) in 20 mM phosphate buffer (pH 6.9) with 6.7 mM sodium chloride were used. COS extract (40 μl) was mixed with 40 μl α-amylase and 80 μl of 20 mM phosphate buffered saline (pH 6.9) and pre-incubated for 15 min at 37 °C. Final concentration of COS extract used was 1 to 6.5 mg/ml. Starch (40 μl) was added after the pre-incubation and the reaction mixtures were incubated for 15 min at 37 °C. Dinitrosalicylic acid colour reagent was added (100 μl) to the tubes and incubated at 85 °C for 15 min. Distilled water (900 μl) was added to the tubes and the absorbance was measured at 540 nm. Appropriate blanks and controls were carried out. Acarbose (Sigma) was used as the standard inhibitor.

### Measurement of α-Glucosidase inhibitory effect of COS leaves

α-Glucosidase inhibitory effect of COS extract was assessed using the method described by Elya et al. [34]. Sodium phosphate buffer (pH 6.8) (200 μl) and 120 μl of 1 mM *p*-Nitrophenyl α-D-Glucopyranoside (Sigma) was added to the tubes. Plant extract (40 μl) was added to the test and test blank. Tubes were pre-incubated for 15 min at 37 °C and then 40 μl of 0.1 U α-glucosidase from *Saccharomyces cerevisiae* (Sigma) was added to the tests and the control. Final concentration of COS extract used was 50 to 100 μg/ml. The reaction mixtures were incubated for another 15 min at 37 °C and the reaction was terminated using 100 mM sodium carbonate (800 μl). Absorbance was measured at 405 nm. Acarbose was used as the standard inhibitor.

### Detection of inhibitory effect of COS leaves on fructosamine formation

Fructosamine formation during the incubation of proteins with sugar was measured using the method described by Meeprom et al. [2] with modifications. Briefly, chicken egg lysozyme (Sigma) was incubated with 500 mM fructose in 200 mM phosphate buffer (pH 7.4) containing 0.02 % sodium azide. Incubation was carried out in the dark in the presence or absence of 250 μg/ml or 5 mg/ml COS extract at 37 °C for 7 days. Aminoguanidine (AG) was used at 1 mg/ml as the positive control. Corresponding blanks were prepared in the absence of fructose. Aliquots were collected at day 5 and analyzed for the reduction of nitroblue tetrazolium. Test samples were mixed with the 0.1 M sodium carbonate buffer (pH 10.35) and left for 5 min. Appropriate blanks were prepared by adding fructose to the test blanks just before the assay. Nitroblue tetrazolium (0.5 M) in 0.1 M sodium carbonate buffer (pH 10.35) was added to the reaction mixtures and incubated at 37 °C for 15 min. Absorbance at 530 nm was measured. Percentage inhibition of the relative fructosamine concentration in the presence of COS and AG was calculated.

### Calculation of percentage inhibition*

Percentage inhibition was calculated using the following formula.

$$ \%\kern0.5em \mathrm{Inhibition}=100-\left[\frac{\left(\mathrm{Absorbance}\ \mathrm{of}\ \mathrm{Test}-\mathrm{Absorbance}\ \mathrm{of}\ \mathrm{Test}\ \mathrm{Blank}\right) \times 100}{\left(\mathrm{Absorbance}\ \mathrm{of}\ \mathrm{Control}-\mathrm{Absorbance}\ \mathrm{of}\ \mathrm{Control}\ \mathrm{Blank}\right)}\right] $$

*Applied the formula to calculate the enzyme inhibition and % inhibition of relative concentration of fructosamine.

### Calculation of IC_50_

The concentration of the extract that inhibits 50 % of the enzyme activity (IC_50_) was measured using a series of suitable extract concentrations. IC_50_ values were determined by plotting percent inhibition (Y axis) versus log10 extract concentration (X axis) and calculated by logarithmic regression analysis from the mean inhibitory values.

### Statistical analysis

Enzyme inhibitory assays and the fructosamine inhibitory assay were performed three times. Each experiment was carried out in triplicates. Statistical analysis was performed using *t*-test. *p* < 0.05 was considered as significant.

### Detection of glycation inhibitory effect of COS leaves

Glycation of bovine serum albumin (BSA) (Sigma) was undertaken in vitro as described by Wijetunge and Perera [35]. In brief, BSA was incubated with fructose (500 mM) in 200 mM phosphate buffer (pH 7.4) containing 0.02 % sodium azide at 37 °C for 30 days. Incubations were conducted in the presence or absence of 1 or 5 mg/ml COS extract. AG (1 mg/ml) was used as the positive control. Corresponding blanks were prepared in the absence of fructose. Aliquots were collected at day 12 or 13 and day 30 and analyzed for the degree of glycation, using polyacrylamide gel electrophoresis (PAGE) under non-denaturing conditions. Electrophoresis was carried out with the Enduro vertical gel electrophoresis system- E2010-P according to the standard Laemmli method using 10 % polyacrylamide gels [36]. Gels were stained with Coomassie brilliant blue. Changes in the migration position of the BSA bands in the aliquots were compared. Approximate percentage inhibition of glycation was assessed in comparison to the uninhibited reaction, based on the decrease in migration of BSA in the presence of COS extract. Experiments were repeated three times.

### Detection of glycation induced protein cross-linking inhibitory effect of COS leaves

Glycation induced protein cross-linking inhibitory effect of COS was assessed using the method described by Perera and Ranasinghe [37]. Briefly, chicken egg lysozyme (Sigma) was incubated with fructose in the presence or absence of 250 μg/ml or 2 mg/ml COS extracts for 14 days. Other conditions were as described in the fructosamine assay and incubated for 14 days. Aliquots were collected at day 6 and 14 and analyzed for the appearance of high molecular weight products using sodium dodecyl polyacrylamide gel electrophoresis (SDS-PAGE). Electrophoresis was carried out with the Enduro Vertical Gel Electrophoresis system- E2010-P according to the standard Laemmli method using 12 % SDS-polyacrylamide gels [36]. Gels were stained with Coomassie brilliant blue. Appearance of high molecular weight products of lysozyme in the aliquots was compared. Experiments were repeated three times.

## Results

Yield of the methanol extract was 15.8 % from the dry COS leaf powder. Dry extract was resuspended in phosphate buffer immediately before the assays.

### α-Amylase inhibitory effect of COS leaves

**Fig. 1:**
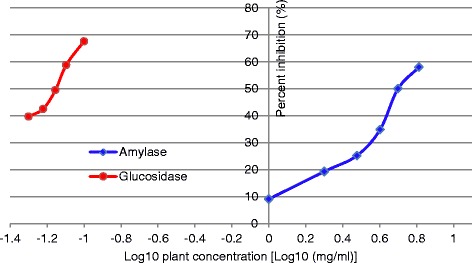
The percent α-amylase and α-glucosidase inhibitions (%) of COS at varying concentrations. Data are indicated as mean percentage inhibition. Final concentrations of the extract used for amylase inhibitory assay were 1, 2, 3, 4, 5, 6.5 mg/ml and for glucosidase inhibitory assay were 50, 60, 70, 80, 100 μg/ml

Even though there was amylase inhibitory effect observed with COS, the IC_50_ for amylase inhibition of COS was 5.88 mg/ml which was significantly higher than the IC_50_ value of the standard inhibitor Acarbose (262.54 μg/ml) for porcine pancreatic amylase (*p* < 0.01). The percent α-amylase inhibitions (%) of COS at varying concentrations are shown in Fig. 1.

### α-Glucosidase inhibitory effect of COS leaves

α-Glucosidase inhibitory effect observed with COS leaves was significantly higher than that of α-amylase inhibitory effects (*p* < 0.01). IC_50_ for glucosidase inhibition of COS was 67.5 μg/ml which was significantly lower than the IC_50_ value of the standard inhibitor Acarbose (208.53 μg/ml) for yeast glucosidase (*p* < 0.01). The percent α-glucosidase inhibitions (%) of COS at varying concentrations are shown in Fig. 1.

### Inhibitory effect of COS leaves on fructosamine formation

**Fig. 2:**
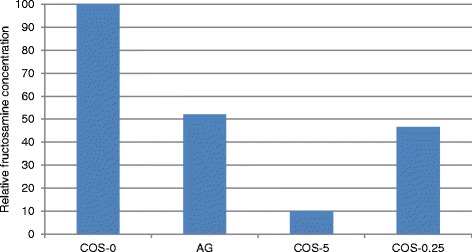
Effect of COS on the formation of fructosamine. Relative concentration of fructosamine formed was considered to be proportionate to the difference between the test (T) and blank (B). T-B of aliquots collected on day 5 of the incubation was compared. T-B of the uninhibited reaction with no extract (COS-0) was expressed as 100 %. COS-0.25: In the presence of 250 μg/ml COS, COS-5: In the presence 5 mg/ml of COS. AG: In the presence of AG

Fructosamine formation was compared using aliquots collected on day 5 of the incubation. Difference between the absorbance of the test and blank is proportionate to the relative concentration of fructosamine present in the aliquot. There was a significant reduction of the relative fructosamine concentration compared to the uninhibited control (*p* < 0.01) with an inhibition of 53.42 % in the presence of 250 μg/ml and 89.95 % in the presence 5 mg/ml of COS extract (Fig. 2). AG showed an inhibition of fructosamine formation by 47.95 % (Fig. 2).

### Glycation inhibitory effect of COS leaves

**Fig. 3:**
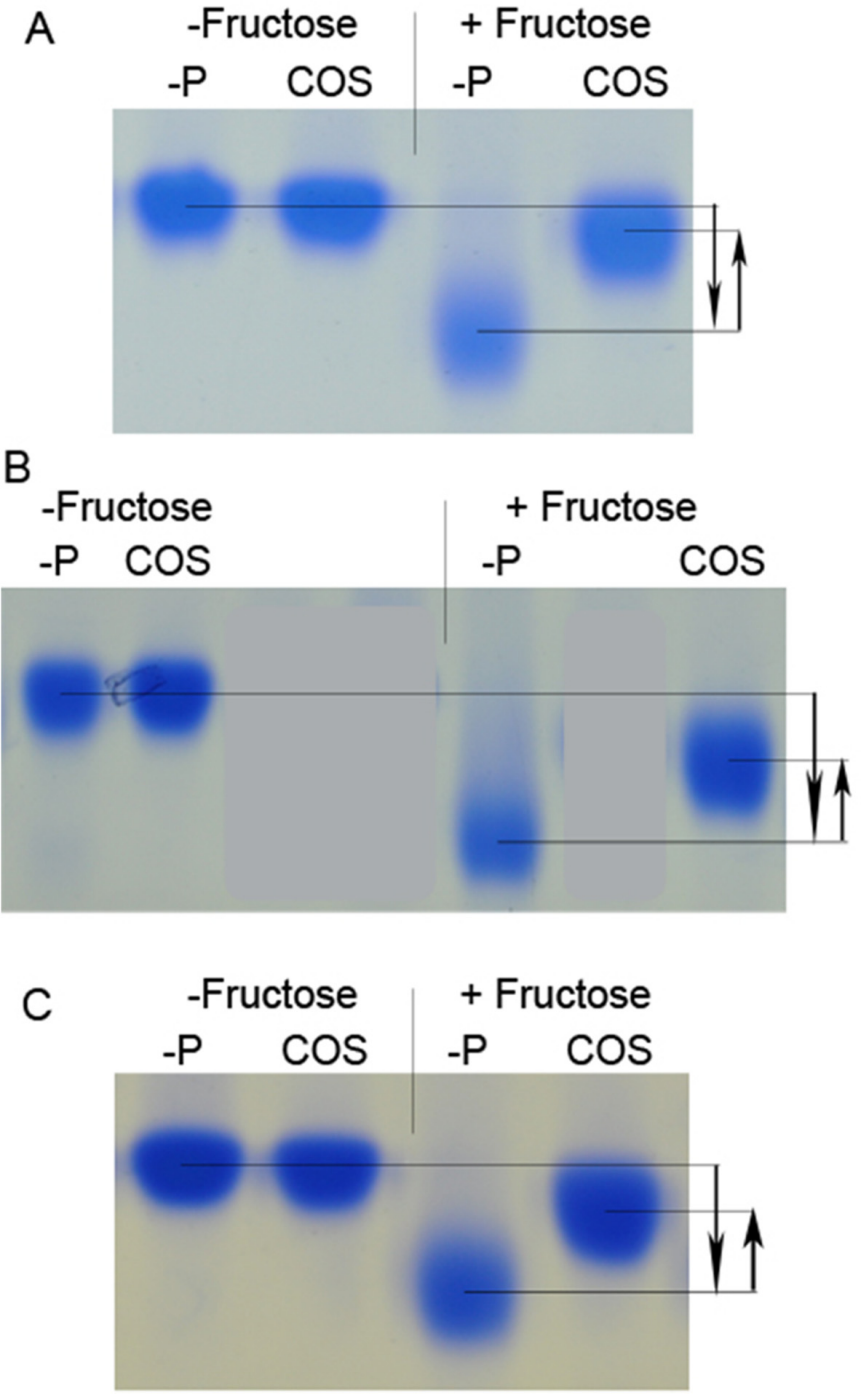
Glycation inhibitory effect of COS. PAGE was conducted. **a**: with aliquots collected on day 12 with 5 mg/ml extracts. **b**: with aliquots collected on day 30 with 5 mg/ml extracts. **c**: with aliquots collected on day 13 with 1 mg/ml extracts. -P: in the absence of COS, COS: in the presence of COS, −Fructose: in the absence of fructose, +Fructose: in the presence of fructose. Experiment was repeated three times

Migration of BSA towards the anode was increased (downward arrow) in the presence of fructose (Fig. 3). Previously we have reported that this increase is proportionate to the degree of glycation [35]. The increase in BSA migration was retarded in the presence of COS (upward arrow) indicating glycation inhibition (Fig. 3). This inhibition was similar to that of the standard inhibitor AG (results not shown). Such a change in migration did not occur in the absence of fructose even when the plant extract (5 mg/ml) was included in the reaction mixture (Fig. 3). Inhibitory effects of COS was observed with both 1 and 5 mg/ml concentrations and the inhibition lasted even on day 30 (Fig. 3). However, the degrees of inhibition seem to reduce with longer incubation and lower concentration of COS, as denoted by the increase in the gap between the height of the two arrows in Fig. 3b and c compared to that of a.

### Glycation induced protein cross-linking inhibitory effect of COS leaves

**Fig. 4:**
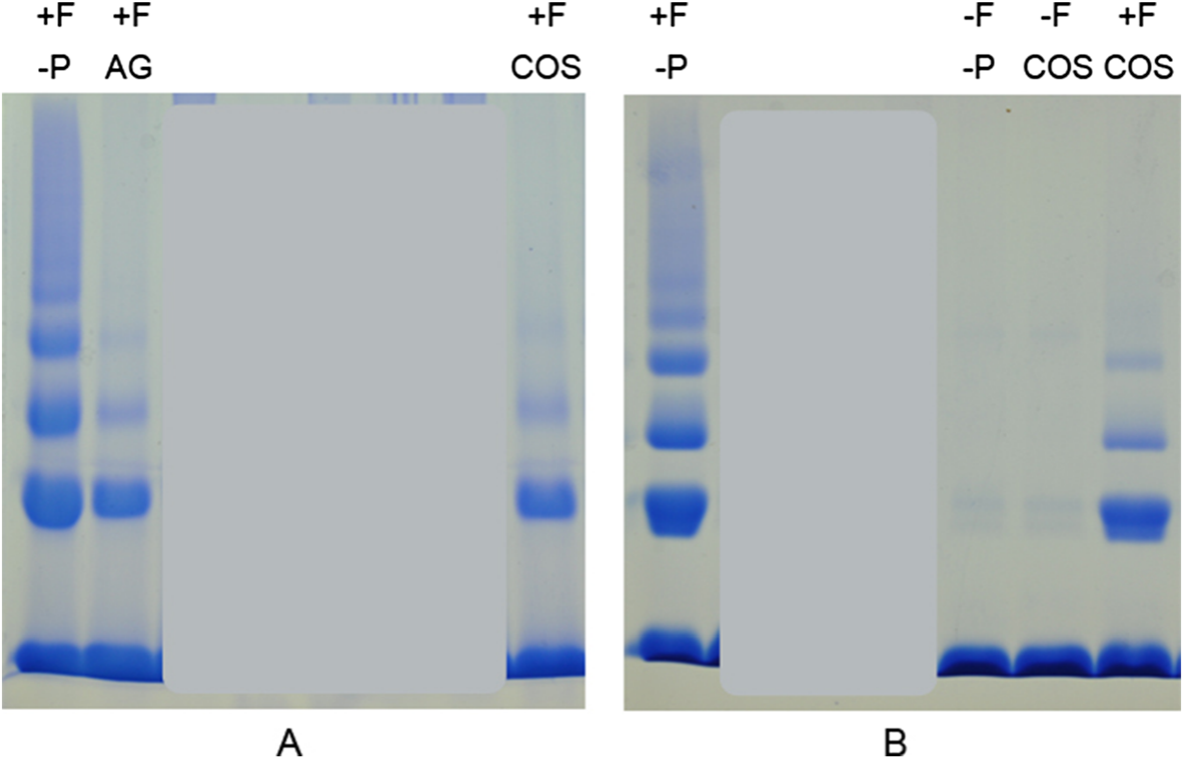
Glycation induced protein cross-linking inhibitory effect of COS. SDS PAGE was conducted. **a**: with aliquots collected on day 14 with 2 mg/ml extract. **b**: with aliquots collected on day 6 with 250 μg/ml extract. -P: in the absence of COS, COS: in the presence of COS, −F: in the absence of fructose, +F: in the presence of fructose. Experiment was repeated three times

High molecular weight products of lysozyme were formed in the presence of fructose (Fig. 4). Previously we have reported that the amount of such products formed is proportionate to the degree of glycation induced protein cross-linking [37]. These products represented the dimer, trimer and tetramer of lysozyme as demonstrated previously using molecular weight markers [37]. There was a reduction in the amount of high molecular weight products formed in the presence of AG and COS leaf extract indicating inhibition of protein cross-linking. The inhibition observed after 14 day incubation with 2 mg/ml COS extract was in parallel with that of AG (Fig. 4a). Inhibitory effect of COS was observed even at a lower concentration (250 μg/ml) of extract (Fig. 4b). High molecular weight products were not detected in the absence of fructose even when COS was included in the reaction mixture (Fig. 4b).

## Discussion

Hyperglycaemia is an independent risk factor in the development of chronic diabetic complications. Therefore the management of type 2 diabetes relies on the maintenance of blood glucose concentration in a normal or near normal level [9]. COS leaves are consumed in the Sri Lankan diet [20–22] and are used to treat diabetes [16, 24, 26]. However, scientific evidence to support the hypoglycaemic effects of COS leaves are lacking. Some plants are known to have glycation inhibitory effects which will provide additional benefit. Antiglycation effects may delay glycation induced diabetic complications even when blood glucose is elevated. As per up to date literature, there are no reports available on the effects of COS leaves on the formation of early or late glycation products. The present study revealed the inhibitory effects of COS leaves on the α-glucosidase, fructosamine formation, protein glycation and glycation induced protein cross-linking.

Several investigations carried out using alloxan or streptozotocin induced diabetic rats have proven the hypoglycaemic effects of COS rhizome. Results of these studies show that COS rhizome increases the insulin secretion and peripheral utilization of glucose. Most of these studies also have shown cholesterol lowering effects of COS. The ethanol extract of COS rhizome showed a significant reduction in blood glucose, glycosylated haemoglobin and increase in liver glycogen and insulin in alloxan induced diabetic rats treated for 60 days (27). Furthermore improvements in many other biochemical parameters such as cholesterol lowering effects were observed in the test group [27]. These effects were comparable with those of hypoglycaemic drug glibenclamide. Ethanol extract of COS roots decreased blood glucose and increased the expression of insulin, insulin receptor, glucose transporter, glucokinase, aldolase, pyruvate kinase, succinate dehydrogenase and glycogen synthase in streptozotocin induced rats treated for 4 weeks [28]. Ethanol extract of COS root significantly reduced blood glucose concentration, increased glycogenesis and decreased gluconeogenesis in alloxan induced rats treated for 4 weeks [29]. Improvement of lipid parameters and hepatic antioxidant enzyme activities were also observed in their study [29]. Petroleum ether, chloroform, methanol and aqueous extracts of COS rhizome were studied in streptozotocin induced diabetic rats on the oral glucose tolerance after a single dose of extracts and the hypoglycaemic effects after multiple doses of extracts for 14 days [30]. Hypoglycaemic effects observed were highest with methanol and water extracts of COS which were in parallel with glibenclamide [30].

Diosgenin is the major constituent isolated from COS [38] and a quantity of 0.37 % was found in leaves [39]. Gavillán-Suárez et al. demonstrated the presence of high content of alkaloids in COS leaves [26]. Among the compounds isolated from COS and other species of genus Costus that have shown hypoglycaemic effects with a concomitant increase in insulin in diabetic rats include diosgenin [40], eremanthin [41], costunolide [42], quercetine glycosides [43] and the pentacyclic triterpene β-Amyrin [44]. Eremanthin isolated from COS rhizome has significantly reduced blood glucose level in a dose dependent manner and glycosylated hemoglobin HbA1c in streptozotocin induced diabetic rats treated for 60 days [41]. Eremanthin has also increased plasma insulin and tissue glycogen while showing hypolipidaemic effects [41]. Costunolide (20 mg/kg) isolated from COS root has significantly decreased glycosylated hemoglobin (HbA1c), total cholesterol, triglyceride, LDL cholesterol, markedly increased plasma insulin, tissue glycogen, HDL cholesterol and serum protein and restored the altered liver enzymes in plasma in streptozotocin induced diabetic rats treated for 30 days [42].

Among the few studies investigating antidiabetic effects of COS leaf, one study reported the effect of COS leaf methanol extract and water extract in reversing the insulin resistance induced by a high fat diet in male Wistar rats treated for 4 weeks [21]. Another study reported the glucose binding capacity and the reduction of glucose diffusion rate with COS leaf extracts in vitro [45]. They also have stated an amylase inhibitory effect of 18 % which was significantly lower than that of Acarbose with 2 % COS leaf using a slightly different method [45]. These findings suggested possible mechanisms of COS leaf extract in delaying the intestinal glucose absorption. A significant association of hypoglycaemia was revealed with the inclusion of COS leaves in the diet in diabetic patients who were on oral hypoglycaemic drugs [24].

Previous findings with COS rhizome showed multiple effects of the extract in the body which can bring down the blood glucose [27]. Effect of the COS on the intestinal absorption of glucose was not reported in these studies except for a very recent study which revealed a marginal amylase inhibitory effect [45]. Even though the amylase inhibitory effects seen in COS leaf is marginal in the current study in agreement with the previous study [45], the current study reveals a significant inhibitory effect of COS on glucosidase activity. Hence COS leaf is likely to blunt the post prandial blood spikes of blood glucose.

Antiglycation effects of *C. speciosus* leaves were not reported as per up to date literature. A few previous studies have shown a significant reduction in glycosylated haemoglobin with eremanthin isolated from COS rhizome for 60 days [41] and costunolide isolated from COS rhizome for 30 days [42] with a reduction in blood glucose. As the specific antiglycation mechanisms were not investigated in these studies, whether the decrease demonstrated in glycosylated haemoglobin was a direct effect of the extract or an indirect effect resulted due to lowering of blood glucose is not clear. It is known that antiglycation effects are correlated with antioxidant activity [46]. There is evidence for antioxidant activity of COS rhizome. Costunolide and eremanthin isolated from the root of COS demonstrated a significant increase in the activity of superoxide dismutase, catalase and glutathione peroxidase when streptozotocin induced diabetic rats were treated for 60 days [47]. Inhibitory effect of *C. pictus* leaves (another species of the genus Costus known as “insulin plant”) on early glycation product fructosamine was assessed using the nitoblue tetrazolium reduction method. Results showed approximately 50 % inhibition of early glycation products by 100 μg/ml methanol extracts of *C. pictus* leaves which was in par with the standard inhibitor AG [48]. We have demonstrated in vitro protein glycation inhibitory effects of COS leaves for the first time, using three methods to monitor inhibition of early as well as late glycation products in the presence of high concentration of sugar. Effect of the COS extract on fructosamine formation at 250 μg/ml observed in the current study matches with the findings of 100 μg/ml *C. pictus* leaves [48]. Whether the inhibition observed in our study on the formation of late glycation products (protein cross-links) is due to the inhibition that occurred on early glycation events or due to inhibitory effects that occur on several stages is not identified. It is understood that a plant with glucose lowering effects will bring down the glycation as a result of the reduction of substrate concentration. However, the methods we adopted are designed to check the inhibitory effects on glycation at high concentration of sugar and therefore are likely to be independent from the hypoglycaemic effects of the extract.

Current study indicates possible mechanisms of COS leaf which may cause hypoglycaemic effects and effects which may delay the chronic diabetic complications in vivo. Even though the methods used are simple, they have been validated for accuracy and reproducibility. However, one limitation of the present study is that the difficulty of making a judgment on the in vivo efficacy purely based on the results of the findings made in vitro. Another limitation is that the safety of the use of COS extracts was not investigated. However, there are no reports on toxic effects of COS leaves and the documented evidence show the resistance to toxic effects caused by substances such as streptozotocin. It was revealed that 50, 100, 150 mg/kg COS leaf water extract cause strong inhibitory effects against the genotoxicity and histopathologic alterations induced by streptozotocin in rats [23]. Even the administration of higher doses (1500 to 3000 mg/kg) of COS leaf aqueous extract orally for 12 weeks did not show features of liver or renal toxicity in insulin resistant rats [22]. Similar evidence on the safety was obtained when the cell viability was measured in cell cultures with methanol extract of COS leaf [49] and ethyl acetate and water extracts of COS leaf [20]. According to the previous literature on the dosage of COS leaf used to lower blood glucose [26], an approximate daily dose of 57.45 mg (~0.82 mg/kg assuming a body weight of 70 kg) COS leaf methanol extract could be suggested to investigate the efficacy in humans. This dosage is far below the dosage used in experimental animals [22, 23].

## Conclusion

The in vitro inhibitory effects of COS leaves on α-glucosidase activity was demonstrated for the first time which may be one mechanism of exerting hypoglycaemic effects of COS leaves in vivo. For the first time the current study reveals the inhibitory effects of COS leaves on the formation of early and late glycation products such as fructosamine and protein cross-links respectively in the presence of high concentration of fructose. These findings provide scientific evidence to support the use of COS leaves for hypoglycemic effects with an added advantage in slowing down protein glycation. Further studies are necessary to evaluate the possibility of using COS leaves as a safe alternative to synthetic antidiabetic drugs.

## Abbreviations

AGEs: advance glycation end products
AG: aminoguanidine
COS: *Costus speciosus*
BSA: bovine serum albumin
SDS-PAGE: sodium dodecyl polyacrylamide gel electrophoresis

## References

[1] International Diabetes Federation (2013). IDF Diabetes Atlas.

[2] Meeprom A, Sompong W, Chan CB, Adisakwattana S (2013). Isoferulic acid, a new anti-glycation agent, inhibits fructose-and glucose-mediated protein glycation in vitro. Molecules.

[3] Sadowska-Bartosz I, Bartosz G (2015). Prevention of protein glycation by natural compounds. Molecules.

[4] Aronson D (2003). Cross-linking of glycated collagen in the pathogenesis of arterial and myocardial stiffening of aging and diabetes. J Hypertens.

[5] Goh SY, Cooper ME (2008). The role of advanced glycation end products in progression and complications of diabetes. J Clin Endocrinol Metab.

[6] Singh VP, Bali A, Singh N, Jaggi AS (2014). Advanced glycation end products and diabetic complications. Korean J Physiol Pharmacol.

[7] Gkogkolou P, Böhm M (2012). Advanced glycation end products: key players in skin aging?. Dermato-Endocrinology.

[8] Li J, Liu D, Sun L, Lu Y, Zhang Z (2012). Advanced glycation end products and neurodegenerative diseases: mechanisms and perspective. J Neurol Sci.

[9] Sheard NF, Clark NG, Brand-Miller JC, Franz MJ, Pi-Sunyer FX, Mayer-Davis E (2004). Dietary carbohydrate (Amount and Type) in the prevention and management of diabetes a statement by the American diabetes association. Diabetes Care.

[10] Mahomoodally MF, Subratty AH, Gurib-Fakim A, Choudhary MI, Nahar Khan S. Traditional medicinal herbs and food plants have the potential to inhibit key carbohydrate hydrolyzing enzymes in vitro and reduce postprandial blood glucose peaks in vivo. The Scientific World J. 2012; doi:10.1100/2012/285284. 10.1100/2012/285284 PMC3361172 22654584

[11] Olaokun OO, McGaw LJ, Eloff JN, Naidoo V (2013). Evaluation of the inhibition of carbohydrate hydrolysing enzymes, antioxidant activity and polyphenolic content of extracts of ten African Ficus species (Moraceae) used traditionally to treat diabetes. BMC Complementary and Alternative Medicine.

[12] Sales PM, Souza PM, Simeoni LA, Magalhães PO, Silveira D (2012). α-Amylase inhibitors: a review of raw material and isolated compounds from plant source. J Pharm Pharm Sci.

[13] Kumar S, Narwal S, Kumar V, Prakash O (2011). α-glucosidase inhibitors from plants: a natural approach to treat diabetes. Pharmacogn Rev.

[14] Grover JK, Yadav S, Vitas V (2002). Medicinal plants of India with antidiabetic potential. J Ethnopharmacol.

[15] Ediriweera ERHSS, Ratnasooriya WD (2009). A review on herbs used in treatment of diabetes mellitus by Sri Lankan ayurvedic and traditional physicians. Ayu.

[16] Jung M, Park M, Lee CH, Kang Y, Kang ES, Kim SK (2006). Antidiabetic agents from medicinal plants. Curr Med Chem.

[17] Modak M, Dixit P, Londhe J, Ghaskadbi S, Devasagayam TPA (2007). Indian herbs and herbal drugs used for the treatment of diabetes. J Clin Biochem Nutr.

[18] Rani AS, Sulakshana G, Patnaik S (2012). *Costus speciosus*, An antidiabetic plant-review. Fons Scientia Journal of Pharmacy Research.

[19] Pawar VA, Pawar PR (2014). *Costus speciosus*: an important medicinal plant. International Journal of Science and Research.

[20] Samarakoon KW, Lakmal HC, Kim SY, Jeon YJ (2014). Electron spin resonance spectroscopic measurement of antioxidant activity of organic solvent extracts derived from the methanolic extracts of Sri Lankan thebu leaves (*Costus speciosus*). Journal of the National Science Foundation of Sri Lanka.

[21] Subasinghe HWAS, Hettihewa LM, Gunawardena S, Liyanage T (2014). Methanol and water extracts of *Costus speciosus* (j.könig) sm. leaves reverse the high-fat-diet induced peripheral insulin resistance in experimental Wistar rats. International Research Journal of Pharmacy.

[22] Subasinghe HWAS, Hettihewa LM, Gunawardena S, Liyanage T (2015). Evaluation of aqueous extract of *Costus speciosus*(J.König)Sm.leaf for hepatic and renal toxicities: biochemical and histopathological perspectives. European Journal of Pharmaceutical and Medical Research.

[23] Girgis SM, Shoman TMT, Kassem SM, Ezz El-Din A, Abdel-Aziz KB (2015). Potential Protective effect of *Costus speciosus* or its nanoparticles on streptozotocin-induced genotoxicity and histopathological alterations in rats. Journal of Nutrition & Food Sciences.

[24] Medagama AB, Bandara R, Abeysekera RA, Imbulpitiya B, Pushpakumari T (2014). Use of complementary and alternative medicines (CAMs) among type 2 diabetes patients in Sri Lanka: a cross sectional survey. BMC Complementary and Alternative Medicine.

[25] Vishalakshi DD, Asna U (2010). Nutrient profile and antioxidant components of *Costus Speciosus* Sm, and *Costus ignes* Nak. Indian Journal of Natural Products and Resources.

[26] Gavillán-Suárez J, Aguilar-Perez A, Rivera-Ortiz N, Rodríguez-Tirado K, Figueroa-Cuilan W, Morales-Santiago L, et al. Chemical profile and in vivo ypoglycemic effects of *Syzygium jambos*, *Costus speciosus* and *Tapeinochilos ananassae* plant extracts used as diabetes adjuvants in Puerto Rico. BMC Complementary and Alternative Medicine. 2015;15:244. doi:10.1186/s12906-015-0772-7. 10.1186/s12906-015-0772-7 PMC4511456 26198986

[27] Revathy J, Abdullah SS, Kumar PS (2014). Antidiabetic effect of *Costus Speciosus* rhizome extract in alloxan induced albino rats. Journal of Chemistry and Biochemistry.

[28] Ali HA, Almaghrabi OA, Afifi ME (2014). Molecular mechanisms of anti-hyperglycemic effects of *Costus speciosus* extract in streptozotocin-induced diabetic rats. Saudi Medical Journal.

[29] Bavarva JH, Narasimhacharya AVRL (2008). Antihyperglycemic and hypolipidemic effects of *Costus speciosus* in alloxan induced diabetic rats. Phytother Res.

[30] Rajesh MS, Harish MS, Sathyaprakash RJ, Shetty AR, Shivananda TN (2009). “Antihyperglycemic activity of the various extracts of *Costus speciosus* rhizomes”. Jof Natural Remedies.

[31] Poongunran J, Perera HKI, Fernando WIT, Jayasinghe L, Sivakanesan R (2015). α-Glucosidase and α-amylase inhibitory activities of nine Sri Lankan antidiabetic plants. British J Pharmaceutical Res.

[32] Geethalakshmi R, Sarada DVL, Marimuthu P, Ramasamy K (2010). α-Amylase inhibitory activity of *Trianthema decandra* L. Int J Biotechnol Biochemistry.

[33] Bernfeld P (1955). Amylases alpha and beta, in Methods in enzymlogy, Volume 1 (Academic Press, New York). Methods Enzymol.

[34] Elya B, Basah K, Munim A, Yuliastuti W, Bangun A, Septiana EK. Screening of *α*-glucosidase inhibitory activity from some plants of Apocynaceae, Clusiaceae, Euphorbiaceae, and Rubiaceae. Journal of Biomedicine and Biotechnology. 2012; doi:10.1155/2012/281078. 10.1155/2012/281078 PMC3236488 22187534

[35] Wijetunge DCR, Perera HKI (2014). A novel in vitro method to detect inhibitors of protein glycation. Asian Journal of Medical Sciences.

[36] Laemmli UK (1970). Cleavage of structural proteins during the assembly of the head of bacteriophage T4. Nature.

[37] Perera HKI, Ranasinghe HASK (2015). A simple method to detect plant based inhibitors of glycation induced protein cross-linking. Asian Journal of Medical Sciences.

[38] Dasgupta B, Pandey VB (1970). A new Indian source of diosgenin (*Costus speciosus*). Experientia.

[39] Srivastava S, Singh P, Mishra G, Jha KK, Khosa RL (2011). *Costus speciosus* (Keukand): a review. Der Pharmacia Sinica.

[40] Naidu PB, Ponmurugan P, Begum MS, Mohan K, Meriga B, RavindarNaik R (2015). Diosgenin reorganises hyperglycaemia and distorted tissue lipid profile in high‐fat diet-streptozotocin‐induced diabetic rats. J Sci Food Agric.

[41] Eliza J, Daisy P, Ignacimuthu S, Duraipandiyan V (2009). Antidiabetic and antilipidemic effect of eremanthin from *Costus speciosus* (Koen.) Sm., in STZ-induced diabetic rats. Chem Biol Interact.

[42] Eliza J, Daisy P, Ignacimuthu S, Duraipandiyan V (2009). Normoglycemic and hypolipidemic effect of costunolide isolated from *Costus speciosus* (Koen ex. Retz.) Sm. in streptozotocin-induced diabetic rats. Chem Biol Interact.

[43] Mosihuzzaman M, Nahar N, Ali L, Rokeya B, Khan AK, Nur EAM (1994). Hypoglycemic effects of three plants from eastern Himalayan belt. Diabetes Research.

[44] Jothivel NPS, Appachi M, Singaravel S, Rasilingam D, Deivasigamani K, Thangavel S (2007). Anti-diabetic activity of methanol leaf extract of *Costus pictus* D. Don in alloxan-induced diabetic rats. Journal of Health Sciences.

[45] Devi VD, Asna U (2015). Possible Hypoglycemic Attributes of *Morus indica* 1. and *Costus speciosus*: An in vitro Study. Malaysian Journal of Nutrition.

[46] Dearlove RP, Greenspan P, Hartle DK, Swanson RB, Hargrove JL (2008). Inhibition of protein glycation by extracts of culinary herbs and spices. J Med Food.

[47] Eliza J, Daisy P, Ignacimuthu S (2010). Antioxidant activity of costunolide and eremanthin isolated from *Costus speciosus* (Koen ex. Retz) Sm. Chem Biol Interact.

[48] Majumdar M, Parihar PS (2012). Antibacterial, anti-oxidant and antiglycatbion potential of *Costus pictus* from southern region, India. Asian J Plant Sci Res.

[49] Nair SV, Hettihewa M, Rupasinghe HP. Apoptotic and inhibitory effects on cell proliferation of hepatocellular carcinoma HepG2 cells by methanol leaf extract of *Costus speciosus*. BioMed Research International. 2014; doi:10.1155/2014/637098. 10.1155/2014/637098 PMC4000957 24818148

